# Fertility Preservation as an Option for Women with Genetic Disorders: Insights from a SWOT Analysis on Elective Oocyte Freezing and Preimplantation Genetic Testing

**DOI:** 10.3390/life13071483

**Published:** 2023-06-30

**Authors:** Greta Chiara Cermisoni, Valerio Pisaturo, Valeria Stella Vanni, Sabrina Minetto, Luca Pagliardini, Rossella Masciangelo, Massimo Candiani, Enrico Papaleo, Alessandra Alteri

**Affiliations:** 1 Obstetrics and Gynaecology Unit, IRCCS San Raffaele Scientific Institute, 20132 Milan, Italy; 2 Department of Maternal and Child Health and Urological Sciences, “Sapienza” University of Rome, Policlinico Umberto I, 00161 Rome, Italy; 3 Reproductive Sciences Laboratory, Obstetrics and Gynaecology Unit, IRCCS San Raffaele Scientific Institute, 20132 Milan, Italy; 4 Department of Brain and Behavioral Sciences, University of Pavia, 27100 Pavia, Italy; 5 Faculty of Medicine and Surgery, Vita-Salute San Raffaele University, 20132 Milan, Italy

**Keywords:** egg freezing, oocyte cryopreservation, fertility preservation, PGT-M, genetic diseases, SWOT analysis

## Abstract

This paper uses a SWOT (strengths, weaknesses, opportunities, and threats) analysis to overview the option of fertility preservation in women with genetic diseases, who would later use preimplantation genetic testing for monogenic disorders, in order to not transmit their condition. Strengths associated with elective oocyte freezing are ethical considerations, overall maternal and fetal safety, and effectiveness, if performed at <35 years of age. Weaknesses are related to costs and rare but present (<1–3%) risks of maternal complications. Counselling on fertility management aimed at preventing infertility offers a valuable opportunity, the same as it has been in oncological patients’ care. The potentially high percentage of women with genetic conditions who would return to use their frozen oocytes also represents an opportunity together with the minimization of the need for egg donation, which has higher obstetrical risks compared to the use of autologous oocytes. Finally, a threat is represented by the potential psychological distress to young women who could never attempt to become pregnant through preimplantation genetic testing, or do it before any decline in their fertility. Potential unknown future long-term health risks for children conceived after egg vitrification/thawing are also a threat, but current knowledge is reassuring. Altogether, early counselling on the option of fertility preservation should thus be incorporated into standard care of all patients with any genetic condition.

## 1. Introduction

The purpose of fertility preservation is to safeguard the reproductive potential of patients whose future fertility is endangered by various medical conditions, such as cancer or other diseases, or by aging. It is well known that female fertility declines with the passage of time and, in this sense, fertility preservation allows women to protect their parental project for possible future realization [1]. A growing body of literature supports a strong association between increasing maternal age and declining oocyte competence and euploidy rates. In addition to deteriorating oocyte quality, rising ovulatory disturbances, diminishing ovulatory frequency, impaired luteal phase, and premature follicle recruitment are other factors that contribute to falling conception rates with advancing reproductive age. Moreover, the postponement of parenthood implies a longer window of time in which lifestyle, pathological, and genetic disruptions can reduce a woman’s chances of conceiving spontaneously. Lifestyle factors that adversely affect fertility can be various, such as smoking, obesity, alcohol use, excessive exercise, and sexually transmitted diseases. All of these can have negative effects on female fertility over the years.

Oocyte cryopreservation practice is a feasible strategy to preserve female fertility potential [2]. Oocyte cryopreservation is considered a major breakthrough in assisted reproductive technology in recent years, with a growing number of women opting to preserve their fertility through this practice, both to delay having children and for medical reasons such as cancer. In this regard, the timing of oocyte retrieval and the number of eggs available for freezing are crucial factors to optimize the cumulative chance of pregnancy with frozen oocytes.

Other patients who could benefit from egg freezing include carriers or patients with genetic diseases. In particular, reproductive consequences of Turner syndrome include primary amenorrhea, premature ovarian insufficiency, and infertility. Very recently, the first live birth achieved using cryopreserved oocytes in a woman diagnosed with mosaic Turner syndrome has been reported, paving an optimistic path for the use of this technique in the future [3]. Other genetic disorders, such as BRCA mutations, may be associated with a low risk of accelerated ovarian aging and a high risk of developing ovarian and breast cancers, suggesting that BRCA carriers have a limited reproductive window and should consider anticipating childbearing or preserving fertility [4,5]. This also applies to patients with sickle cell disease [6], galactosemia [7], and others [5], for whom an accelerated age-associated decline in ovarian reserve is demonstrated. Nevertheless, not all genetic disorders affect reproductive potential.

Oocyte collection and in vitro fertilization, however, also allows preimplantation genetic testing for monogenic diseases (PGT-M), which identifies embryos that will be affected by the disease in order to transfer only those without the mutation. Currently, PGT-M can be used to detect more than 600 genetic conditions, and its use is rapidly increasing [8].

### Preimplantation Genetic Testing for Monogenic Diseases (PGT-M)

Interest in PGT-M for affected individuals/carriers of single-gene disorders is explained by two main crucial domains, namely (i) avoidance of the suffering of an affected child and of the termination of an affected pregnancy [9] and (ii) economic concerns related to raising an affected child. It is documented that having a child with a genetic disease can use as much as over 30% of a family’s income [10]. Moreover, a recent US cost-effectiveness study showed that PGT-M for Huntington’s disease is not only a successful strategy to relieve morbidity and mortality in offspring, but it also reduces costs compared to spontaneous conception which is followed by the use of prenatal genetic testing [11].

While this evidence supports the employment of PGT-M in patients with genetic diseases, awareness about their reproductive prognosis in assisted fertilization cycles is still scarce. Van Der Keler and colleagues have recently published data from a study of 2265 PGT-M cycles involving couples with monogenic diseases not associated with reduced ovarian reserve or ovarian response. The authors showed that the study population has a lower reproductive prognosis in terms of cumulative live birth rate (CLBR) than infertile couples who do not perform PGT for intention-to-treat, despite a younger age. Importantly, this effect was observed in the absence of any known impact of the genetic disease on female ovarian function. While the overall prognosis of PGT-M remains good (CLBR 29.4% compared to 35.0% in non-PGT-M couples), [12], this implies that a significant number of couples may be required to undergo multiple IVF cycles to achieve a successful conception of a healthy offspring. This finding confirms previous smaller studies in which a fit-to-transfer blastocyst rate of 34.5% was found in couples undergoing PGT-M [9]. As a matter of fact, the proportion of non-transferable embryos after PGT-M varies from 25 to 81% of screened embryos, depending on the type of genetic inheritance characterizing the disease and the genotype of each partner [13]. In line, a median number of inseminated oocytes as high as 27 has been reported to obtain a median number of 2 unaffected, euploid embryos [9].

However, even with as few as two retrieved oocytes or a single embryo available for transfer, it is possible to predict a cumulative live birth rate (CLBR) of over 10% in PGT-M cycles [12]. This suggests that the efforts involved in PGT-M are overall justified if the couple is willing to not transmit the disease.

In this context, the strategy of oocyte cryopreservation at a young age would prevent the detrimental effects of the reduced ovarian reserve and the age-related increase in aneuploidy and would allow the storage of a high number of oocytes and therefore blastocysts for PGT-M.

**Figure 1:**
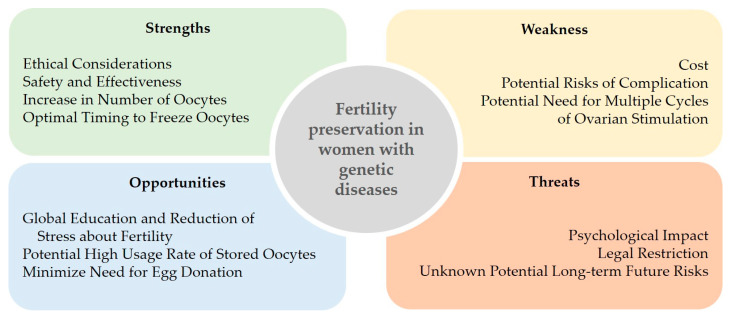
SWOT Analysis of fertility preservation in women with genetic diseases.

On these bases, an analysis of strengths, weaknesses, opportunities, and threats (SWOT analysis) was conducted to overview oocyte cryopreservation for women who are at high risk of having offspring affected by genetic diseases (Figure 1).

## 2. Strengths

### 2.1. Ethical Considerations, Safety, and Effectiveness

The Ethics Committee of the American Society for Reproductive Medicine found elective egg freezing to be ethically permissible based on the main arguments of enhancing reproductive autonomy and promoting social equality [14].

Additionally, avoidance of suffering of an affected child and avoidance of termination of an affected pregnancy [9] seem to contribute to the ethical admissibility of PGT-M.

Safety issues such as potential harm to the embryos and short- and long-term health problems for the baby have been identified as the main concern for couples with monogenic diseases in relation to assisted reproduction [15]. Importantly, the egg vitrification technique has, instead, largely demonstrated its safety and efficiency. To date, vitrified oocytes have a significantly high survival rate after warming. Studies have shown that the reproductive efficacy of vitrified oocytes is comparable to that of fresh oocytes in terms of fertilization, embryo aneuploidy, implantation, clinical pregnancy rates, and live birth rate [16,17]. In addition, reassuring evidence comes from studies evaluating the safety of oocyte vitrification in oocyte donation programs [18,19,20,21,22] and in fertility preservation patients [23], showing no differences in obstetric and neonatal outcomes between vitrified versus fresh oocytes.

### 2.2. Increase in Number of Oocytes

Oocyte freezing could therefore raise the number of oocytes available for those women who, due to their risk of transmitting a genetic disorder to their children, intend to proceed with preimplantation diagnosis in the future to obtain a healthy baby. This strategy has already been applied to build larger cohorts of available oocytes [24,25], and to help increase the number of euploid blastocysts in the preimplantation genetic testing cycle [26,27]. In PGT-M cycles, the number of inseminated oocytes is positively associated with the probability of having a transferable embryo and with clinical outcome [12,28].

### 2.3. Optimal Timing to Freeze Oocytes

High rates of live births can be achieved when egg freezing is performed at a young age, approximately before the age of 38. Literature however shows a lack of agreement on the optimal time for cryopreservation of oocytes. Some studies show that the highest probability of achieving successful births from cryopreserved oocytes is before the age range of 36–38 years [29,30]. Other studies and a meta-analysis suggest that the optimal age for oocyte cryopreservation should preferably be before the age of 35 years [31,32]. Taken together, the data thus suggest that the ‘best case scenario’ for oocyte freezing is below age 36 [8].

## 3. Weaknesses

### 3.1. Cost

The cost of elective oocyte freezing can vary depending on several factors, including the geographical location, the clinic or fertility center chosen, and any additional services or medications required. In most countries, non-medical fertility preservation is not supported by public health systems and is expensive. Financial issues also play a strong role in patient decision-making. This means that this strategy is not available to all women, exacerbating existing inequalities and raising concerns about equity, as resources would be diverted from solving other fertility issues that are, by contrast, more evenly distributed across the population. These considerations highlight the complex nature of incorporating elective freezing into public reimbursement schemes. Healthcare systems must carefully evaluate the potential benefits and drawbacks, taking into account issues of equity, cost-effectiveness, and the broader context of fertility care in patients with genetic disorders.

### 3.2. Potential Risks of Complication

It is not always clear what constitutes as a medical reason for fertility preservation, and some argue that this represents an unnecessary medicalization of women, bearing in mind the minimal, but real, risks of ovarian stimulation and ovarian retrieval. One of the most frequent consequences of ovarian stimulation is ovarian hyperstimulation syndrome (OHSS) [33]. Mild to moderate forms of OHSS are medically manageable and can be resolved within a few days. Severe to critical OHSS involves massive enlargement of the ovaries, pleural effusion, acute renal insufficiency, hemoconcentration, and thromboembolic phenomena. These complications are potentially life-threatening. Fortunately, with the implementation of ovarian stimulation protocols that use antagonist and agonist triggers, the risk of developing OHSS is greatly reduced. To date, it occurs in 1–3% of in vitro fertilization cycles. In addition, women should be well aware of the possibilities of the other main complications of ovarian retrieval, such as infections, organ damage, blood loss, and ovarian torsions during egg retrieval, which are all estimated to be below 1–3% [34,35,36].

### 3.3. Potential Need for Multiple Cycles of Ovarian Stimulation 

Women with a compromised ovarian reserve may need several controlled ovarian stimulations and ovarian retrievals to obtain a favorable number of oocytes. Multiple cycles of oocyte accumulation can be costly and can potentially have a physical and psychological impact. This might apply, for example, to patients with sickle cell disease [6], galactosemia [7], and others [5], for whom an accelerated age-associated decline in ovarian reserve is demonstrated. However, oocyte accumulation in low-responder patients has proven to be an effective strategy in in vitro fertilization cycle, with a lower drop-out rate and increased cumulative live birth rates compared with using fresh samples after each stimulation [37]. A similar approach could be needed in some patients with genetic conditions.

## 4. Opportunities

### 4.1. Global Education and Reduction in Stress about Fertility

There is a need to improve fertility education for the general public. Many women report having received information about elective egg freezing mostly from friends and non-medical sources [38]. When information on this topic is not given by healthcare professionals, there is a risk of diffusion of false optimism and false myths. Fertility preservation counselling in women affected by monogenic disorders is less often a primary concern than it is in oncological patients [39]. The provision of comprehensive fertility care in light of medical evidence is emerging as an obligation for physicians of patients with genetic diseases [40]. Discussion of elective egg freezing options is advised at the earliest for females with a monogenic disorder. This would allow them to make informed personal choices based on (i) the patient’s family plan; (ii) the effect of age, individual risk factors, and lifestyle factors on reproductive outcome; (iii) the impact of monogenic disease on fertility; (iv) the prognosis of a future IVF cycle with PGT-M with a possible future partner; and (v) the opportunity to cryopreserve oocytes and the timing of the procedure.

### 4.2. Potential High Usage Rate of Stored Oocytes

One of the main criticisms directed at elective egg freezing for non-medical reasons is a low cost–benefit ratio, and also due to a low usage rate of previously frozen oocytes. The largest published study reports that only 12.1% of women return to use their oocytes [41]. This is mostly due to the spontaneous conception of these women. In contrast, women with a monogenic disorder who have decided to cryopreserve their oocytes will most likely turn to an assisted reproduction center to select the healthy embryo in which the target genetic condition is absent. The potential higher usage rate in this group of women remains, however, purely speculative and there are currently no data or reports on this topic.

### 4.3. Minimize the Need for Egg Donation

European statistics indicate a growing demand for donated oocytes in assisted reproductive technology. This highlights the escalating need for donated oocytes and the importance of addressing this demand to support couples undergoing fertility treatments in Europe [42]. The use of cryopreserved oocytes at an early age may represent an autologous oocyte donation, in terms of success rates, considering a normal chromosome set. This procedure, in the most common sense, could diminish the women’s need for heterologous donation and allow for genetic children when they feel ready.

## 5. Threats

### 5.1. Psychological Impact

Discussing the potential of elective egg freezing with women with genetic disorders, who have never thought about family issues due to their young age or lack of a partner, could encourage the patient to further postpone pregnancy, which increases the risk of maternal and neonatal complications [43]. In some cases, it could potentially further distress young women who may never try to become pregnant and who may never experience infertility. In any case, it is the task of the healthcare professionals to inform the population about reproductive health so that they can make informed choices.

### 5.2. Legal Restriction

The laws that regulate patients’ access to the procedure vary from country to country. For example, there are European countries where this procedure is forbidden, others where it is allowed in accordance with some medical conditions, and some others where it is also allowed without any clinical indication. Similarly, different states in the USA have different legislations regarding fertility preservation and relative insurance coverage [44]. Furthermore, there may also be different national regulations regarding the maximum period for which these biological samples can remain stored [14].

### 5.3. Unknown Potential Long-Term Future Risks

Many studies reported very good results in terms of survival and clinical outcomes after thawing vitrified oocytes, as we have already discussed above. Nevertheless, there are currently no data about the survival rate and clinical outcomes of oocytes affected by or carrying monogenic mutations. Moreover, these could also be very heterogeneous due to the heterogeneity that exists between the various monogenic disorders. Finally, although it appears reassuring that no major obstetrical and perinatal risks have been found in pregnancies carried out with frozen oocytes so far, these risks cannot be completely ruled out [18,19,21].

## 6. Practical Issues and Patient Counseling for Fertility Preservation in Monogenic Disorders

Fertility counseling should be an integral part of the management of monogenic disorders. Unfortunately, very often, the discussion of the genetic disorder’s impact takes place when the woman begins to contemplate her parental plan, thus more frequently at an advanced age and potentially too late to intervene effectively [45]. Moreover, the potential impact of fertility is usually discussed in cases where there is a direct impairment of ovarian function. It is worth emphasizing how important it is to discuss the risks, even in cases of monogenic disorders not known to be associated with reduced ovarian reserve or response, in light of the recent evidence discussed above.

In fact, family planning remains poorly discussed. Among pediatric and adult nephrologists involved in autosomal dominant polycystic kidney disease care, only 20–40% reported informing their patients about the available pre-implantation diagnostic options [46].

Not surprisingly, the awareness and attitude regarding fertility preservation have never been investigated among professionals caring for patients affected by monogenic diseases.

On the patients’ side, to the best of our knowledge, a survey regarding fertility preservation in non-oncologic genetic diseases is only available for female patients with cystic fibrosis [47], among whom 74% reported never having had conversations about fertility preservation with their healthcare providers.

Even for genetic diseases leading to premature ovarian insufficiency, no specific recommendations concerning fertility preservation are available [5], and the subject is only recently receiving consideration [40].

At the present stage, it seems therefore advisable for the professionals involved to refer patients to reproductive healthcare experts, in order to ensure adequate counseling and management through a multidisciplinary approach.

As a matter of fact, as it is still very challenging to outline what qualifies as a medical reason to cryopreserve oocytes, women need to be informed about the pros and cons of elective egg freezing by a fertility preservation team, ideally consisting of a gynecologist, an embryologist, a psychologist or counselor, and a geneticist. This review aims at providing useful information to be shared with patients in this context.

## 7. Prenatal Diagnosis: When Patients Do Not Choose PGT-M

Non-invasive prenatal diagnosis (NIPD) of monogenic disorders has been developed thanks to the presence of cell-free fetal DNA that is detectable in the maternal circulation, and it can be offered to couples at known increased risk without the associated miscarriage risk that accompanies invasive prenatal testing [46].

Detection of paternally inherited and de novo mutations has become quite uncomplicated, and several techniques have been improved to be used in the clinical setting, but a greater challenge is represented by the detection of maternally inherited mutations due to the abundant presence of maternal cell-free DNA [48].

The spectrum of monogenic disorders that can be tested for by using NIPD includes cystic fibrosis, spinal muscular atrophy, and Duchenne and Becker muscular dystrophies. Nevertheless, many monogenic conditions are relatively rare, and therefore often a custom-made and disease-specific detection method is necessary for rare familial variants. In addition, these tests need to be worked up prior to pregnancy in order to be appropriately validated and therefore usable at the correct timing of pregnancy to aid in decision-making. The testing is technically challenging, making it less readily available for commercialization and diffusion in the clinical setting [49].

Despite its undoubted usefulness, NIPD is still surrounded by some concerns in the case of rare monogenic diseases, such as the unawareness about critical issues regarding incomplete penetrance and variable expressivity, and the absence of large-scale validation and patient follow-up. This ultimately leads to patients making decisions about their pregnancy based on incomplete information.

Moreover, expanding the procedure to low-risk pregnancies might increase detection of variants of uncertain or unknown significance, with no clear clinical interpretation, which challenges patient counseling [48].

Most importantly, when a mutation or a gene involved in a specific disease is detected, couples must be informed about the risks and alternatives of either continuing or interrupting the pregnancy. Even if therapeutic interruption of pregnancy might have positive outcomes, especially for patients with strong distress caused by the diagnosis of fetal malformation, it undoubtedly has important consequences on the mental and reproductive health of women, as it carries a great traumatic burden and psychopathological effect [50].

## 8. Future Perspectives on Fertility in Women with Genetic Diseases

Monogenic causes of female infertility have increased dramatically over the last few decades. Hundreds of genes have been identified, and the clinical validation of just as many gene–disease relationships has been evaluated [51]. In addition, for several known genetic mutations, a possible detrimental effect on ovarian reserve has been poorly investigated, as in the case of over twenty non-BRCA mutations in the BRCA pathway [52].

Advancements in this field will imply moving towards personalized reproductive medicine, with an emphasis on molecular genetic diagnostics and, hence, prevention. In addition, the literature shows that even patients carrying a disease with an unknown association with female fertility (to date, in Europe, we know of 600 diseases for which we perform PGT-M) still have a lower prognosis in terms of cumulative live birth rate compared to non-PGT couples [12]. In this context, elective egg freezing, which allows more young oocytes to be cryopreserved, could become increasingly useful. This is precisely what is needed to improve the prognosis in women who have the risk of transmitting a monogenic disease to their offspring [12].

## 9. Limitations of the Present Work

The main limitation of our analysis is that it conveys information that might not be widely applicable to all clinical contexts. As a matter of fact, relevant technical difficulties exist for both oocyte cryopreservation and PGT-M, and a very specific counselling is required for both, as outlined by current guidelines [53,54]. Patients should thus be referred in a timely manner to tertiary care IVF centers with wide experience in oocyte vitrification/thawing and in embryo biopsy and PGT-M. However, not all patients and clinicians might have access to this option.

## Data Availability

Not applicable.
